# Implications for surveillance for breast cancer patients based on the internally and externally validated BRENDA-metastatic recurrence score

**DOI:** 10.1007/s10549-023-06898-z

**Published:** 2023-03-14

**Authors:** Florian Ebner, Jessica Salmen, Davut Dayan, Matthias Kiesel, Regine Wolters, Wolfgang Janni, Achim Wöckel, Manfred Wischnewsky

**Affiliations:** 1 Universität Ulm, Prittwitzstr. 43, 890 Ulm, Germany; 2 Gyn-Freising, Marienplatz 5, 85354 Freising, Germany; 3 Universitätsfrauenklinik Würzburg, Würzburg, Germany; 4 FB Mathematik u. Informatik, Universität Bremen, Bibliothekar. 1, 28359 Bremen, Germany

**Keywords:** Breast cancer, Follow up, Prognostic, Recurrence, Prediction

## Abstract

**Purpose:**

Although the incidence of distant relapse is decreasing, 20–30% of patients with early breast cancer die of metastasis. The aim of this study is to characterize patients with metastasis-free survival(MFS) less than 5 years, to analyze the most probable site of metastases according to the internally and externally validated BRENDA-score. The BRENDA-score is a combination of the biological subtype and clinical staging.

**Method:**

3832 patients with primary diagnosis of breast cancer and either distant metastatic recurrence within 5 years or MFS ≥ 5 years were assigned to this study. Patients were classified for metastatic recurrence according to the BRENDA-score. 1765 patients were in a validation set. Statistical methods were Kaplan–Meier curves, Cox regression analysis, Exhausted CHAID, likelihood-ratio tests and the Nearest Neighbor Estimation method.

**Results:**

There was a significant(p < 0.001) difference between the Kaplan–Meier MFS-functions of M0-patients stratified by BRENDA-score. The BRENDA score outperforms intrinsic subtypes and the Nottingham prognostic score. It fits the original data and the validation set equally well (p = 0.179).There was a significant(p < 0.001) difference between mean BRENDA-Index for patients with MFS < 5y(21.0 ± 9.0) and patients with MFS ≥ 5y(mean BRENDA-Index 11.7 ± 8.2). 55.6% of the very high risk patients(BRENDA-Index ≥ 27) had metastases within 5 years. The most likely primary metastatic site was bone(30%) followed by liver(19%) and lung(18%). The discriminatory ability(areas under the time dependent ROC curve) of the BRENDA score is good to acceptable for the first 5 years. In the very low/low risk (intermediate, high/very high) risk group 50% of all metastases were diagnosed within 26 months. Guideline adherence had a highly significant influence on outcome independent of the risk group.

**Conclusion:**

The evaluation showed that the BRENDA-Score is a robust predictive tool for breast cancer recurrence and site of metastases in the first five years after diagnosis. It outperforms intrinsic subtypes and the Nottingham prognostic score. The BRENDA-score could be a tool for a risk orientated and targeted follow up.

## Introduction

In Germany the recommended follow up regime is clinical visits at 3 months intervals in the first three years, followed by two years with check ups every 6 months and then returning to an annual check up schedule. This recommendation is not based on prospective trials and tumor biology and survival after recurrence is improving [1, 2]⁠. Even though it is known that the pattern of distant metastasis depends on the tumour biology [3–5], primary treatment and tumor TNM. The follow up recommendations are still ‘one standard fits all’.

Several publications have investigated the clinical orientated follow up versus more intense follow ups (i.e. including further imaging technology like MRI or tumor markers after initial therapy (surgery, chemo- and/or radiotherapy)) [1, 6]⁠⁠. Thus far the optimal interval, methods and parameters have not been determined by prospective randomised trials. This might be due to the different health systems, cost and benefit considerations, available resources or the missing survival benefit of an earlier, smaller tumor detection [7]⁠.

The publication of Wu et al. [5] describes the metastatic pattern of the breast cancer subtypes. Their publication did not consider the tumor stage as another possible predictor for the metastatic site. To include this information the BRENDA database was used to create a Metastatic Reccurrance Score (BRENDA-Score). This score combines the intrinsic subgroups with the clinical staging into one score. Based on this score patients can be classified according to the risk of general metastasis (high/medium/low/very low risk) over the first five years after diagnosis. In this analysis the score is combined with the intrinsic subtype of the tumor to identify the organs at risk in this time frame. Ideally enabling the clinician to screen the organ just before/at its highest risk for a recurrance and detect the recurrance as small/early as possible.

The questions this combination of the BRENDA-Score and intrinsic subtypes should answer are:

Within the first five years:

- When are most patients with a high/very high BRENDA score diagnosed a metastasis and in which organ?
- Can the prediction of the organ and time be improved by combining the BRENDA score with the intrinsic subtypes?
- What would be the adaptation for the clinical follow up?

## Materials and methods

The BRENDA database (BRENDA breast cancer care under evidence-based guidelines) has been used for several epidemiologic breast cancer studies [8–10]. In this retrospective multicenter cohort study, data from the University of Ulm and 16 partner clinics (all certified breast cancer centers) in Baden-Wuerttemberg (Germany) between 2000 and 2008 was analysed.

This database included information on extract TNM-stage, histologic subtype, grading, lymphatic and vascular invasion, estrogen/progesterone/erbB-2-expression, date of diagnosis, and all adjuvant therapies. Data on adjuvant therapies, including surgery (date of surgery, BCT breast-conserving surgery, mastectomy, sentinel-node-biopsy, and axillary lymph node dissection), adjuvant systemic chemotherapy, adjuvant endocrine therapy, and adjuvant radiotherapy, were collected. The quality of these data is considered high [11]⁠. Written and informed consent was obtained from all patients included in this clinical study. The inclusion criterion was histologically confirmed invasive breast cancer and observation time for metastasis-free survival of more than 5 years after primary diagnosis. The latter inclusion criterion indicates that patients either metastasized within the first 5 years or had a metastasis-free survival of more than 5 years.

The exclusion criteria were carcinoma in situ, primary metastatic disease, bilateral breast cancer, primary occult disease, phyllodes tumor, and patients with incomplete follow-up.

### Intrinsic subtypes

To define the intrinsic breast cancer subtypes hormone receptor expression (HR), HER2 expression and cell proliferation marker Ki67 are generally used [12]⁠. As Ki67 was not available in the BRENDA database, we used grading as a surrogate parameter to include the cell proliferation, as described before [13, 14]. Further details on the relationship between grading, KI-67 and intrinsic subtypes are published [15, 16]⁠. The 5 intrinsic subtypes are defined as follows: Luminal A (HR + /HER2 − /grade1 or 2), luminal B-HER2-negative like (HR + /HER2 − / grade 3), luminal B-HER2-positive like (HR + /HER2 + , all grades); HER2-overexpressing (non-luminal, HR − /HER2 +) and triple-negative (basal-like, HR − / HER2 −).

## Metastatic Recurrence Index (BRENDA-Index) and Score (BRENDA-Score)

As previously published [17]⁠ a mulivariate Cox regression analysis was performed to identify significant predictors for metastasis-free survival. This model includes intrinsic subtypes, tumour size, grading, and nodal status as “baseline” predictors. The BRENDA-Index derived from the Cox regression model is defined as follows:

BRENDA-Index = 5*luminal B-HER2-negative like + 4*luminal B-HER2-positive like + 7*HER2-overexpressing + 8*triple-negative + 5*T2 + 9* T3/T4 + 4*G2 + 6*G3 + 8* nodal status(1 ≤ N ≤ 3) + 15*nodal status(N > 3).

The values of the various predictors are either 1 (if yes) or 0 for all other cases. The BRENDA-Index (range 0–38) was divided into five risk groups (very low ≤ 4; low 5–14; intermediate 15–21; high 22–26 and very high-risk ≥ 27) by using exhausted chaid for 5 year metastasis-free survival. These groups define the metastasis recurrence score (BRENDA-Score). The BRENDA-Index and BRENDA-Score were internally and externally validated. The percentages of metastatic recurrence in the first 5 years after diagnosis were 2% (very low group), 5%, 10%, 18% and 30% (very high risk group).

### Statistical analysis

Patient characteristics were described with percentages, mean values and standard deviations (SD). When no information was available, the status was coded as missing data. Statistical comparisons for categorical data are carried out using the χ2 test. The distribution of a continuous parameter across a binary variable was tested using the independent-samples Mann–Whitney U test. Metastasis-free survival (MFS) is the length of time from the start of treatment for cancer that a patient is still alive and the cancer has not spread to other parts of the body. Survival distributions and median survival times are estimated using the Kaplan–Meier product-limit method. The log-rank test was used to compare survival rates. The Cox proportional hazards model was used to estimate the hazard ratio and confidence intervals. Proportional hazards were tested for all entered variables using statistical and graphical methods (Schoenfeld residuals and log–log plot of cumulative hazard). Confidence intervals for the regression coefficients are based on the Wald statistics. We compared the goodness of fit of two nested Cox regression models (e.g. BRENDA score vs Nottingham prognostic score or intrinsic subtypes) with a likelihood-ratio test based on the ratio of their likelihoods. In order to show how well the BRENDA score predicts the one- to five- year recurrence free survival we used the Nearest Neighbor Estimation (NNE) method of Heagerty, Lumley and Pepe [18]⁠. NNE creates time-dependent ROC curves from censored survival data for various time points of interest. The NNE method guarantees in contrast e.g. to the Kaplan–Meier method that sensitivity and specificity were monotone in X for the bivariate distribution function of (X, T), where T represents survival time. In order to test the accuracy, quality and generalizability of this prediction model this model was validated externally with a cohort of 1765 patients (primary diagnosis between 2005 and 2015). P-values less than 0.05 were considered statistically significant. Statistical analyses were two sided and carried out using R 4.1.2, SPSS 28 (IBM) and NCSS 10.

## Results

3832 patients with primary diagnosis from 2000 onwards and either distant metastatic recurrence within 5 years (n = 628; 16.4%) or metastatic free survival ≥ 5 years (n = 3204; 83.6%). were assigned to this study (Table 1; < 5 years vs >  = 5 years). 145 (3.8%) patients were M1. The median age was 62 years (range: 26–89 years). The median tumor size was 2.0 cm (range: 0.1–20.0 cm). 4.7% (*n* = 182) of the patients had T3/T4 stage tumor. 59.1% (*n* = 2264) were luminal A, 16.2% (*n* = 621) luminal B Her2-negative, 9.6% (*n* = 368) luminal B HER2-positive, 5.2% (*n* = 200) HER2 over-expressing and 9.9% (*n* = 379) triple-negative. Furthermore 40.2% (*n* = 1542) were nodal-positive and 31.9% (*n* = 1222) G3 (Table 1).

**Table 1:** Basic characteristics of the two databases used (BRENDA and Dachau)

BRENDA database		Total	MFS < 5 years	MFS ≥ 5 years	Sig.
Variables		n = 3832	628 (16.4)	3204 (83.6)	
Age		61.0±12.5y [26 - 89y]	62.8±13.7y [26 - 89y]	60.6±12.2y [27 - 89y]	< 0.001
Tumor size					
T1		2088 (54.1)	187 (9.0)	1901 (91.0)	< 0.001
T2		1562 (40.8)	357 (22.9)	1205 (77.1)	
T3/T4		182 (4.7)	84 (46.2)	98 (53.8)	
Nodal status					
Nodal negative		2290 (59.8)	177 (7.7)	2113 (92.3)	< 0.001
1-3 affected lymph nodes		891(23.3)	172 (19.3)	719 (80.7)	
> 3 affected lymph nodes		651 (17.0)	279 (42.9)	372 (57.1)	
Grading					
G1		320 (9.4)	19 (5.9)	301 (94.1)	< 0.001
G2		2290 (59.8)	291 (12.7)	1999 (87.3)	
G3		1222 (31.9)	318 (26.0)	904 (74.0)	
Intrinsic subtypes					
Luminal a		2264 (59.1)	238 (10.5)	2026 (89.5)	< 0.001
Luminal B HER2 -		621 (16.2)	145 (23.3)	476 (76.7)	
Luminal B HER2+		368 (9.6)	85 (23.1)	283 (76.9)	
HER2 overexpressing		200 (5.2)	61 (30.5)	139 (69.5)	
Triple negative		379 (9.9)	99 (26.1)	280 (73.9)	

Dachau database		Total	MFS < 5 years	MFS ≥ 5 years	sig.
Variables		n = 1765	156 (8.8)	1609 (91.2)	
Age		59.2±11.1y [31 - 90y]	60.4±13.7y [32 - 90y]	58.6±10.8y [31 - 90y]	< 0.001
Tumor size					
T1		1065 (61.5)	70 (44.9)	1015 (63.1)	< 0.001
T2		392 (22.2)	65 (41.7)	327 (20.3)	
T3/T4		53 (3.0)	14 (9.0)	33 (2.4)	
Others (Tis, TX, etc)		235 (13.3)	7 (4.5)	228 (14.2)	
Nodal status					
Nodal negative		1142 (64.7)	60 (38.5)	1062 (67.2)	< 0.001
1-3 affected lymph nodes		480 (27.2)	45 (28.8)	435 (27.0)	
> 3 affected lymph nodes		143 (8.1)	51 (32.7)	92 (5.7)	
Grading					
G1		190 (10.8)	3 (1.9)	187 (11.6)	< 0.001
G2		1335 (75.6)	114 (73.1)	1221 (75.93)	
G3		240 (13.6)	39 (25.0)	201 (12.5)	
Intrinsic subtypes					
Luminal A		1278 (72.4)	81 (51.9)	1197 (74.4)	< 0.001
Luminal B HER2 -		96 (5.4)	16 (10.3)	80 (5.0)	
Luminal B HER2+		183 (10.4)	25 (16.01)	158 ( 9.8)	
HER2 overexpressing		70 (4.0)	8 (5.1)	62 (3.9)	
Triple negative		138 (7.8)	26 (16.71)	112 (7.0)	

**Fig. 1:**
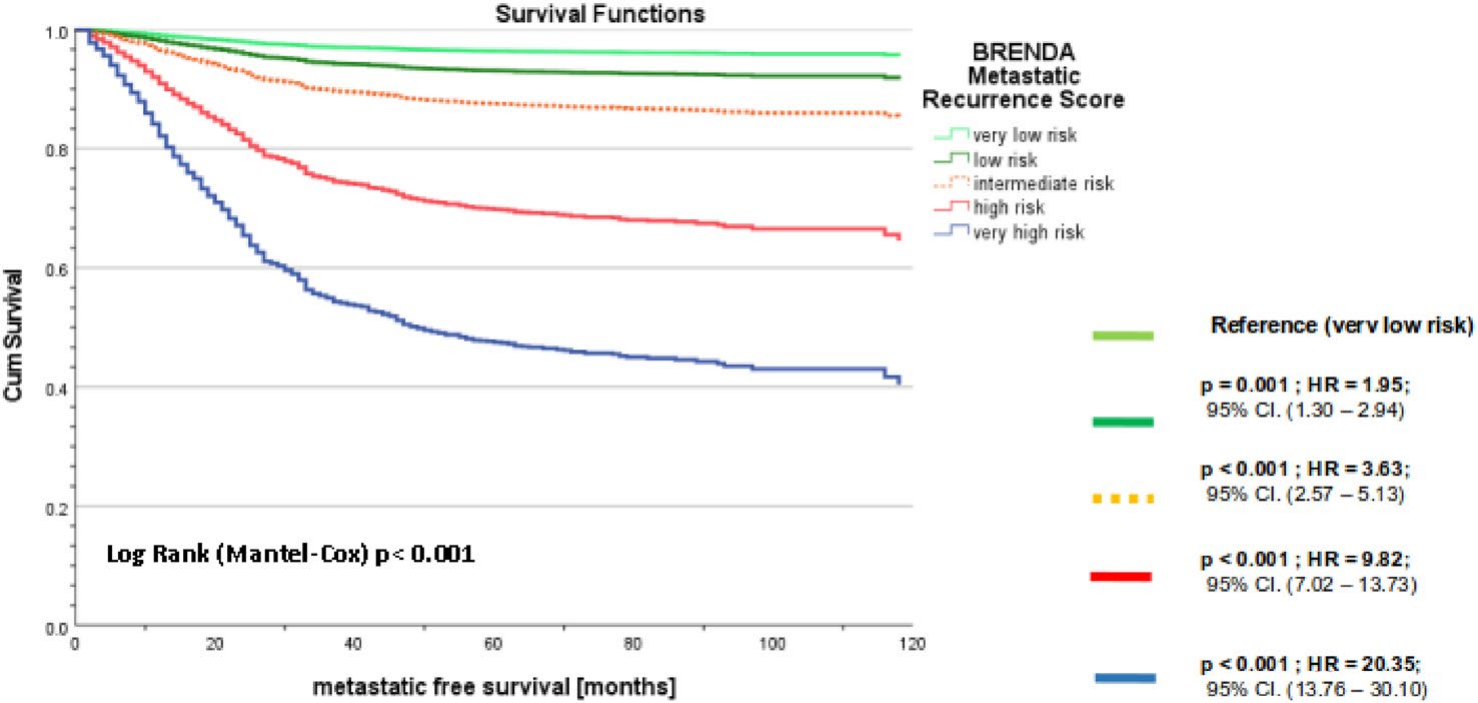
Kaplan–Meier curves of metastasis-free survival of M0-patients (n = 3687) stratified by BRENDA-Risk Score

There was a highly significant difference between the metastasis-free survival functions of M0-patients stratified by BRENDA-risk score (Fig. 1). In numerous publications, intrinsic subtypes were key predictors for metastasis-free and overall survival. The intrinsic subtypes are part of the BRENDA risk score, i.e. can be interpreted as “nested model” of the BRENDA score (full model). The hypotheses are now:

**Fig. 2:**
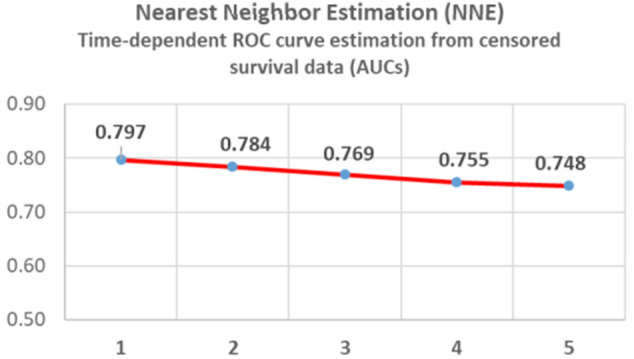
Areas under the curve of time-dependent ROC curves for variuos time points (1-5 years)

**Fig. 3:**
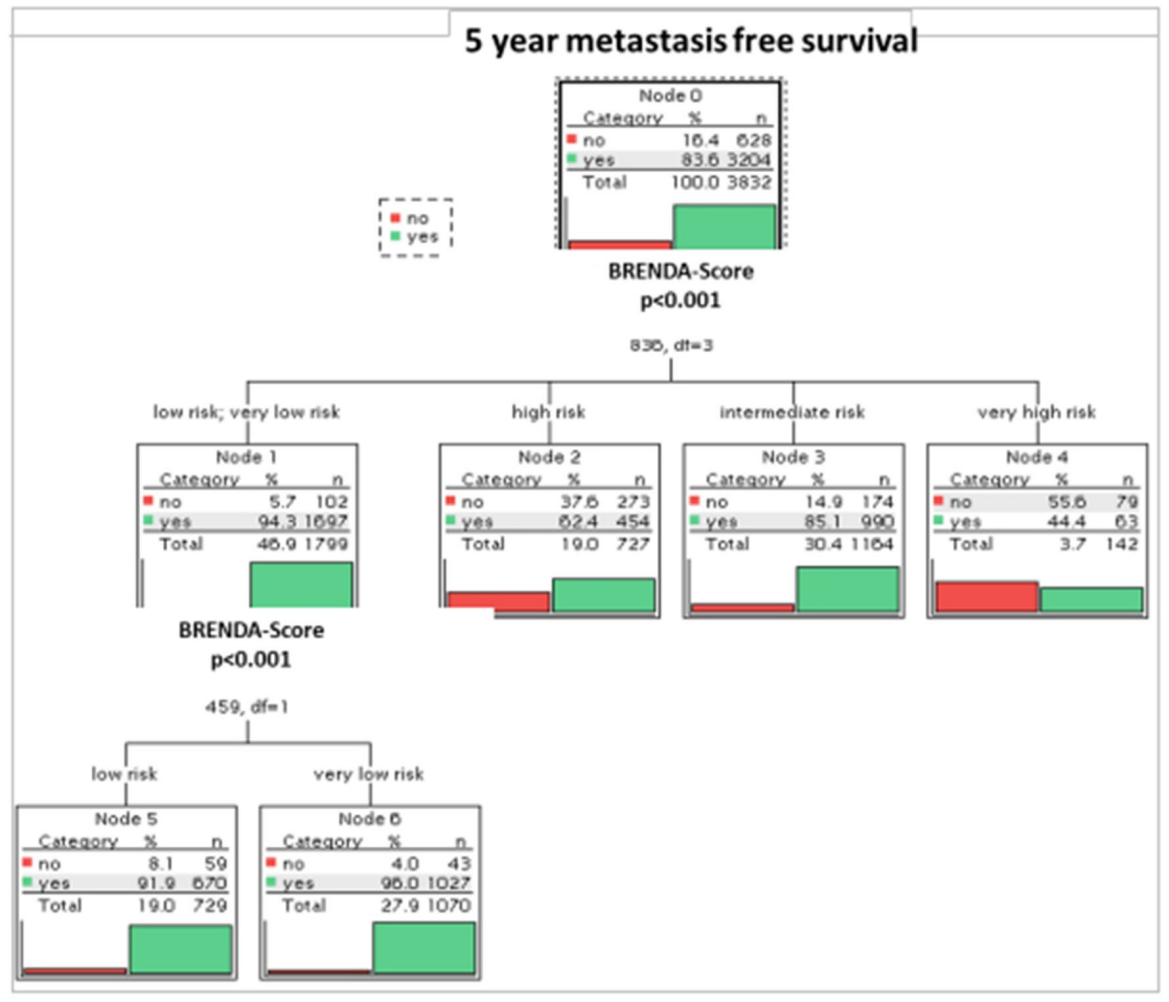
Exhausted Chaid decision tree of year metastasis-free survival and BRENDA-Risk Score

**Fig. 4:**
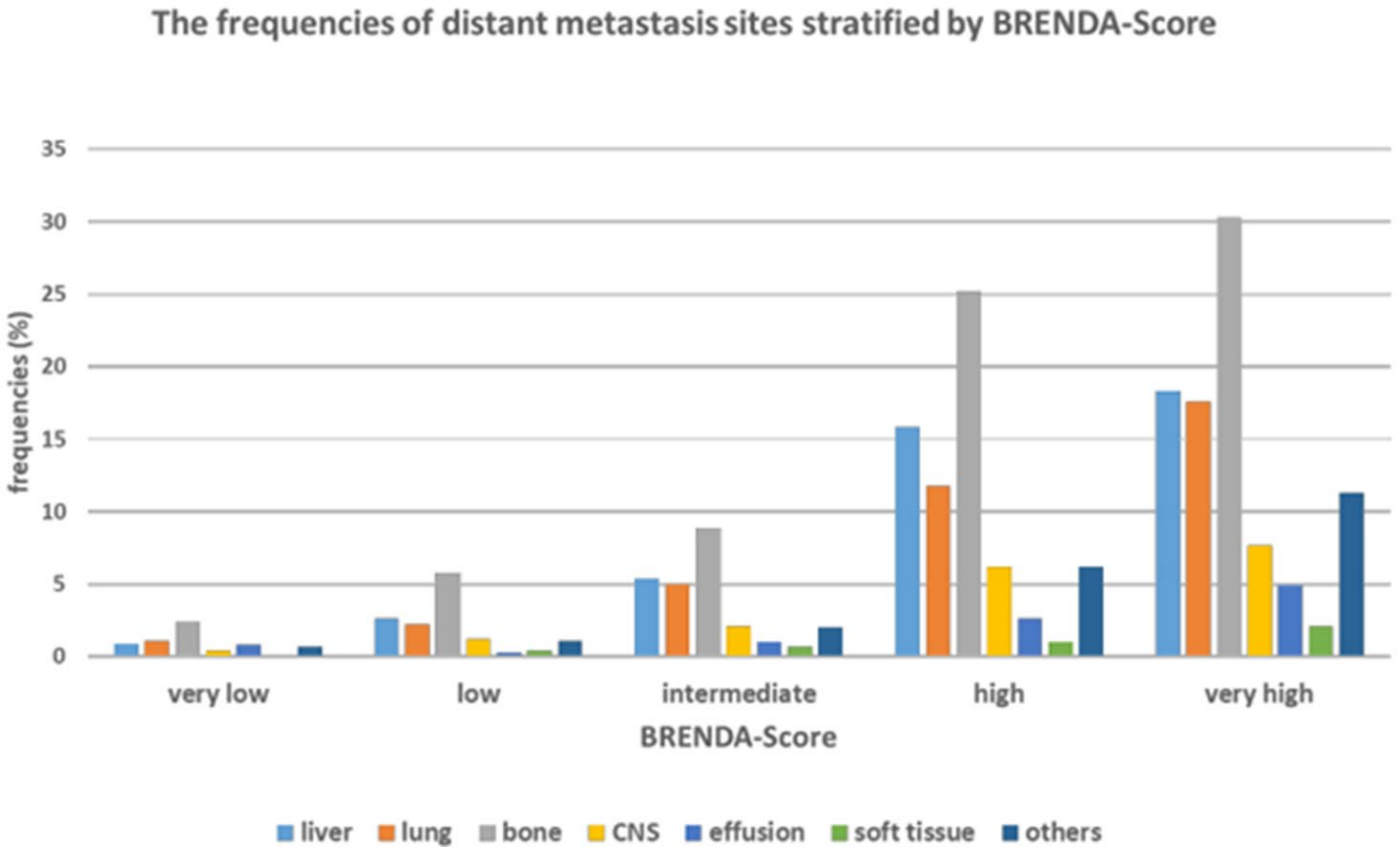
The frequencies of distant metastases stratified by BRENDA-Risk Score

**Fig. 5:**
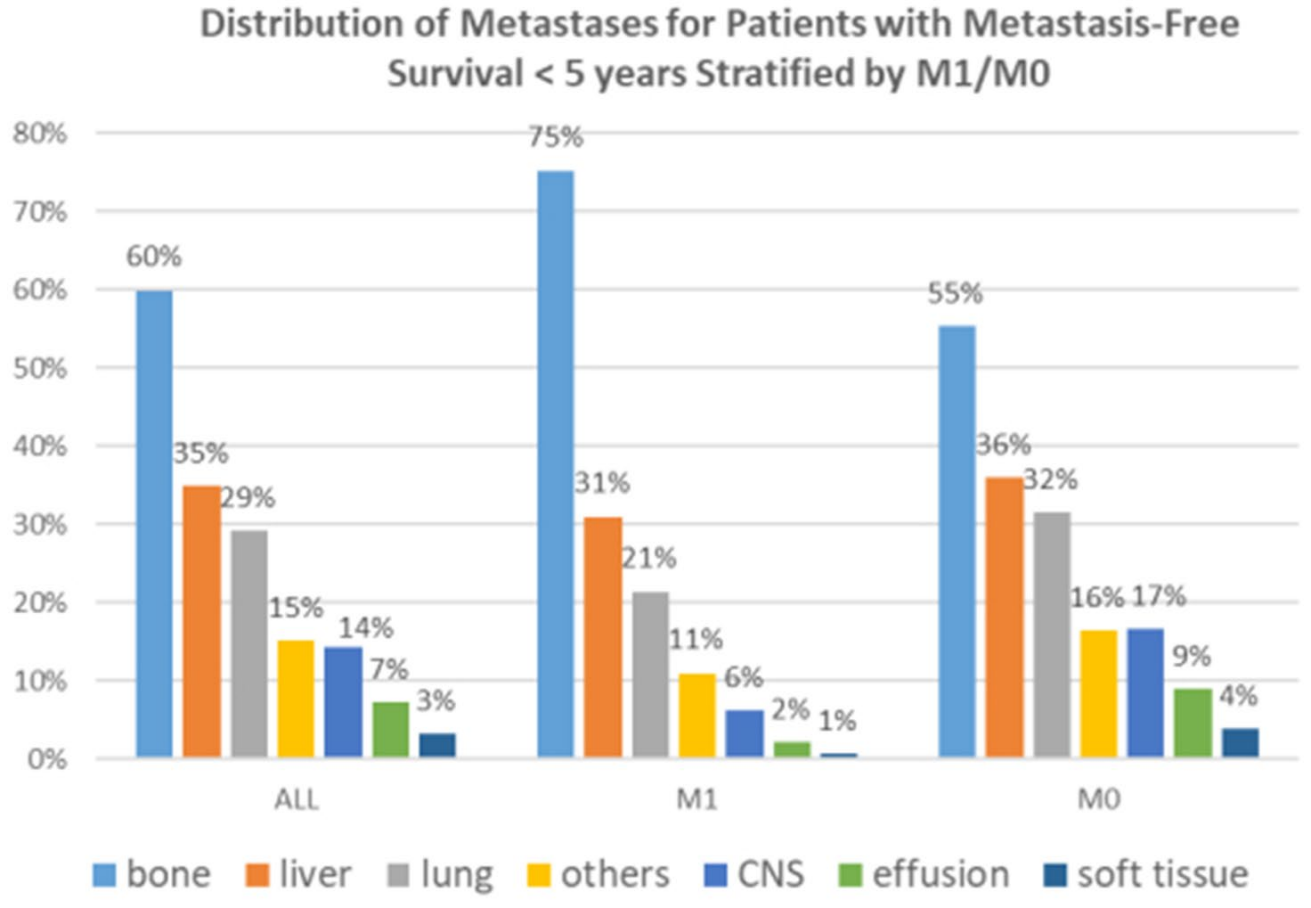
Distribution of metastases of patients with metastasis-free survival stratified <5 years by M1/M0 at primary diagnosis

**Fig. 6:**
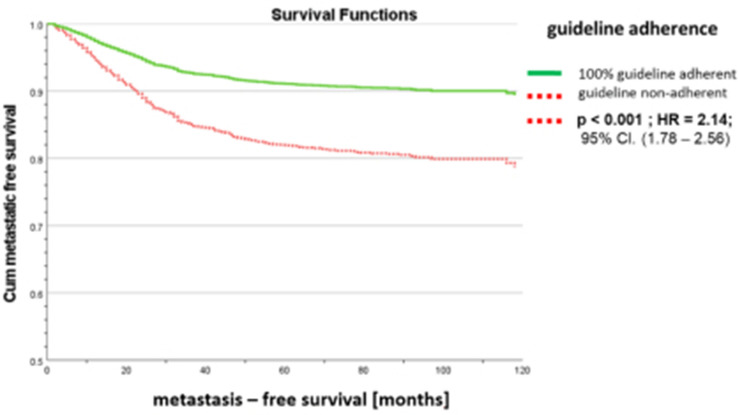
Metastasis-free survival of all M0-patients stratified by guideline-adherence

Null hypothesis **H0**: Both the BRENDA score and the intrinsic subtypes fit the data equally well. As a result, we should employ intrinsic subtypes as model (this model is simplier) and alternative hypothesis **H1**: The BRENDA score significantly outperforms the intrinsic subtypes in terms of data fit. As a result, we should use the BRENDA score.

Since the p-value of a likelihood ratio test comparing the two corresponding Cox regression models is < 0.001, we can reject the null hypothesis and conclude that the BRENDA score significantly outperforms the intrinsic subtypes model. If we take the Nottingham Prognostic Score (NPS; another well established prognostication tool in the management of breast cancers taking tumor size, nodal stage and tumor histological grade into consideration) instead of intrinsic subtypes we obtain the same result: The BRENDA score significantly outperforms NPS. As a result, we should employ the BRENDA score.

External validation of prognostic models is imperative to determine a prediction model’s reproducibility and generalizability to new and different patients. The BRENDA-score was internally and externally validated [17]⁠. We evaluated the BRENDA score again externally for the patient collective in this paper with a data set of 1765 patients from another clinic (Dachau breast center – details Table 1). Again, the null hypothesis could not be rejected (p = 0.179) i.e. the BRENDA score fits the data of the original data set and the evaluation data set equally well (Table 2). Table 3 shows the basic results of the corresponding Cox regression model of the validation set.

**Table 2:** Cox regression model of metastasis-free survival of M0-patients stratified by BRENDA risk Score (reference: very low risk) with hazard ratios, 95% CI, Wald statistics and significance

Variables in the Equation								
	B	SE	Wald	df	Sig	HR	95.0% CI for HR	
							Lower	Upper
BRENDA-Score*			380.2	4	< 0.001			
Low risk	0.67	0.21	10.37	1	0.001	1.95	1.3	2.94
Intermediate risk	1.29	0.18	53.54	1	< 0.001	3.63	2.57	5.13
High risk	2.28	0.17	178.3	1	< 0.001	9.82	7.02	13.73
Very high risk	3.01	0.20	227.7	1	< 0.001	20.4	13.76	30.1

**Table 3:** Cox regression model of metastasis-free survival of 1765 M0-patients in the validation set stratified by BRENDA risk Score (reference: very low risk) with hazard ratios, 95% CI, Wald statistics and significance

Validation set of 1765 patients								
	B	SE	Wald	df	p-value	HR	95.0% CI for HR	
							Lower	Upper
BRENDA-Score*			139.02	4	< 0.001			
Low risk	1.18	0.24	24.84	1	< 0.001	3.27	2.05	5.20
Intermediate risk	1.08	0.20	28.70	1	< 0.001	2.95	1.99	4.38
High risk	2.24	0.21	115.51	1	< 0.001	9.36	6.22	14.07
Very high risk	2.90	0.41	49.00	1	< 0.001	18.09	8.04	40.69

The predictive performance of the BRENDA score of the recurrence free survival time for the patients in the dataset is investigated. In particular, we want to see how well the BRENDA score predicts the one to five year recurrence free survival. We computed time-dependent ROC curves from recurrence free survival data for the various time points using Nearest Neighbor Estimation (NNE) method [18]. The values for the areas under the ROC-curves are given in Fig. 2. AUCs are an effective way to summarize the overall diagnostic accuracy of a test. The discriminatory ability (0.797 – 0.748) is good to acceptable.

Patients with metastasis-free survival ≥ 5 years had a highly significantly (p < 0.001) lower mean BRENDA- Metastatic Recurrence Index (mean 11.7) compared to patients with metastasis-free survival < 5 years (mean 21.0) (Table 4). This result was controlled with the following decision tree (observation time > 5 years). Figure 3 shows a highly significant (p < 0.001) dependence between 5 year metastasis-free survival and BRENDA- Risk Score. Looking at the recurrence risk within the first 5 years the percentage of patients with metastases increases with BRENDA-Score from 4.0% for very low risk patients up to 55.6% for very high-risk patients.

**Table 4:** Mean BRENDA Metastatic Recurrence Index for patients with metastasis-free survival < 5 years and ≥ 5 years

Metastatic free survival	N	Mean	Std. Deviation	Std. Error	95% Confidence Interval for Mean		p-value
					Lower Bound	Upper Bound	
< 5 years	628	21.0	9.0	0.4	20.3	21.7	< 0.001
≥ 5 years	3204	11.7	8.2	0.1	11.5	12.0	
Total	3832	13.3	9.0	0.1	13.0	13.6	

Next the metastases within 5 years were further analysed. Figure 4 shows the percentage of the various distant metastases per BRENDA-risk Score group. For patients with metastasis-free survival < 5 years the most common primary locations of metastases were bone (60%), liver (35%) and lung (29%) followed by others (15%) and CNS (14%) (Fig. 5). This includes all primarily metastasised patients. Therefore two subgroups according to the primary M-classification were formed. The ranking of the metastatic sites did not change between the subgroups. As the BRENDA score is determined by the tumor biology, size and lymph nodes the question arouse what effect has guideline adherence (GA) treatment on metastasis-free survival. The statistical analysis of M0-patients showed a significant difference in survival between the two groups. GA is an independent highly significant predictor for metastasis-free survival (Fig. 6). So in order to show the differences in the BRENDA subgroups the M0-patients with a survival of < 5 years were stratified according to MFS, GA and BRENDA Score (Table 5). The results show a difference of MFS of up to 13.4 months (intermediate risk group).

**Table 5:** Mean metastasis-free survival of M0-patients with metastases within 5 years stratified by BRENDA-Score and guideline adherence

BRENDA Metastatic Recurrence Score						Guideline-adherence	Mean MFS	Std. Error	95% Confidence Interval	
									Lower Bound	Upper Bound
Very low/low risk						Guideline non-adherent	23.0	2.1	18.9	27.1
						Guideline adherent	34.2	1.9	30.4	38.0
Intermediate risk						Guideline non-adherent	18.9	1.3	16.3	21.6
						Guideline adherent	32.3	1.9	28.6	36.0
High/very high risk						Guideline non-adherent	17.8	1.0	15.9	19.7
						Guideline adherent	24.4	1.5	21.5	27.3

The final step in our analysis aimed at determining the time of diagnosis of the metastases and the metastatic site. Therefore the median (and 90% percentile) MFS per metastatic site were calculated for the M0-patients with metastases within 5 years after primary diagnosis according to the recurrence sites. The results according to the BRENDA score classification are provided in Table 5. In the high/very high risk group liver metastasis were on median (90% percentile) diagnosed at 13 (40.8) months. The corresponding overall values for these patients were 18 (38) months, for bone metastases 20 (34.0) months, for lung 14 (33) and for CNS 15 (43) months. In the very low/low risk BRENDA group 50% of all metastases were diagnosed within 26 months. Again the single organs varied between 19 (36.8) months (liver) and 29 (55.0) months for lung metastasis.

## Discussion

The initial treatment of breast cancer has diversified over the last decades. The detection of recurrences in the follow up period has not adapted to the biological tumor knowledge. The national guidelines initially recommend a clinical exam every three months for two to three years, followed by bi-annual exams/visits and then after 5 year annual controlls. Mammography and breast ultrasound should be used alternatingly once a year [19]⁠. Unfortunately there are no prospective randomized trials, nor trials showing a benefit for more intensive follow up examinations [19]⁠. This may be due to the heterogeneous disease which breast cancer is and the varying metastasis pattern. Various models have been published on the initial prognosis and optimal treatment [, , 20–22]⁠. Including regular extended imaging technology [23, 24]⁠⁠ or lately using liquid biopsy [25)]. Some authors have focused on sing^21^, ^26^–^28^]⁠ and published easy to use nomograms to estimate the risk of liver, brain or lymphnode involvment.

The prediction model for liver metastases of Lin et al. [29] is based on 6200 metastasised patients, similar evaluation methods like our score. The areas under the curve (ROC) is given with 0.66 and 0.65 for the training and validation. Interestingly the authors recommend in the discussion using our BRENDA database to improve their nomogram. As the BRENDA database contains more detailed information on tumor and patients than the SEER database which was used in the Lin et al. model. Takada et al. created a model for CNS recurrences using 776 patients with HER2 + breast cancer with neoadjuvant treatment. The AUC values were 0.785 and 0.871 DFS respective brain metastasis [30]. Using 128 patients with brain metastasis Graesslin et al. created a nomogram for any breast cancer subtype with an AUC 0.68 and 0.74 in the validation and training set [31]. So both models are well evaluated for different subgroups of patients providing a likelyhood of CNS metastasis. Graesslin et al. simulate a prophylactic brain radiation to prevent CNS metastasis for patients with a risk greater than 24%. In lung cancer prophylactic radiation results in a survival benefit [32, 33]⁠. An estimation model for bone metastasis has been published in 2015 [34]⁠. The multivariate analysis of just over 300 bone only metastasis patients resulted in a risk estimation at 3,5,7 and 10 years. This was evaluated with an external database and showed a concordance index of 0.73. Though based on a very big database the authors mention as limitation undetected asymptomatic bone metastasis in the routine follow up, possible bias due to unknown bisphosphonate use in osteoporosis and special subgroups that may be underrepresented (like the complete pathological response patients under neoadjuvant systemic treatment). Compared to our model the main differences are the longer prediction time and use of fewer detailed variables.

The models for liver and CNS recurrences provide only a general risk over time. The bone model is split into risks in the 3rd, 5th, 7th and 10th year. Currently the clinician has to use at least three different models to give the patient a detailed risk assessment. So patients at risk can be identified but the clinical consequence is yet to be determined in breast cancer. For example the Dutch influence nomogram [27, 35]⁠ estimates the risk of local recurrence per year. This helps the clinician to make sure the affected breast/thoracic wall is examined carefully at the high risk period. A suspected local recurrence is easy to be clinically examined, imaged and sampled. The treatment options range from resection, radiotherapy to anti-hormonal or systemic treatment. This shows the variety of possible nomograms for patients and caretakers. Our BRENDA Score on the other hand targets not only one organ. Its risk classification comes with the most likely place and time for metastases from an ex ante viewpoint. This will help patients and care takers to tailor a follow up plan acording to the tumor stage and intrinsic subtype as both are included in the score calculation.

In Denmark the policy towards the follow up has recently changed [36]⁠. Patients have open access to the clinic. And Saeltbek et al. report that 15% of the recurrences get diagnosed on patient requested appointments. This is were the BRENDA-Score could help in scheduling and planing. Our results show for the organs at risk (liver, lung, bone and brain) the median (and 90%) time of diagnosing a recurrence in the first 5 years of follow up. Now clinicians could adapt the follow up appointments to the times at risk. And at these appointment attention should be payed on the organ at risk. Though the BRENDA-Score seems to be a reliable tool and the clinical impact could be tremendous there are however weaknesses that need to be addressed.

The variable that is not considered in the BRENDA-Score is the guideline adherence of the treatment. The impact of guideline adherent treatment has been published for several subgroups by various study groups [3, 9, 10]⁠. A current review showed moderate evidence for the impact of guideline adherent treatment on breast cancer patients in general [37]. Retrospectively the guideline adherent treatment is easily included. From an ex ante perspective of the clinician this might be an argument towards the patient to proceed with the recommended treatment. Still there will be the patients who discontinue with the prescribed medication or have to stop due to side effects. But this might not always be known to the follow up clinician. Thus the BRENDA-Score might overestimate the individual recurrence risk. So there is the possibility of improving the score by including the actual finished treatment but then again guideline adherent treatment includes often a long term treatment like anti-hormonal treatment for 60 months. In this period most of the metastases get diagnosed. So follow up appointments based on possible higher recurrence rates will not lead to an underdiagnosis of metastases.

Another weakness is the missing information if earlier detection does enable more successful treatment of the recurrence. Thus far more intense follow up has not been transferred in longer survival [19, 38–40]⁠. Unfortunately previous studies did not correlate the survival to the diameter of the metastases. The primary focus was on the general earlier diagnosis with more follow up appointments or diagnostic tools. Sopik et al. for example [41] looked into predicting the survival after recurrence. The authors published a difference between ER + and ER- recurrences and survival. But did not record the diameter of the recurrence. For patients with a HR + /Her2- recurrence multiple metastasis effects the survival [42]. But the study database was submitted by physicians. So there is the risk of a selection bias. Anyhow this could indicate that an earlier diagnosis with only single metastasis might be beneficial for this subgroup of patients.

But this is where the BRENDA-Score is a starting point by identifying patient subgroup at a very high risk for recurrence and ‘predicting’ the organ and median time of diagnosis of the recurrence. This reduces unnecessary screening imaging in low risk breast cancer patients. By focusing on the organs at the highest risk and possibly monitor this organ more intensively (i.e. blood sample or imaging) a higher rate of detection can be concluded. This theoretic individualisation of the follow up needs to be evaluated for its patients and health care cost benefit in prospective trials (Table 6).

**Table 6:** 50% (median) and 90% percentile of metastasis-free survival stratified by BRENDA-Metastatic Recurrence Score

BRENDA-Metastatic Recurrence Score						MFS: median (90% percentile)				
						All	bone	liver	lung	CNS
Very low/low						V	27 (50.0)	19 (36.8)	29 (55.0)	25 (46.5)
Intermediate						21.5 (46.7)	19 (48.9)	19.5 (53.5)	23 (47.1)	23 (46)
High/very high						18 (38.0)	20 (34.0)	13 (40.8)	14 (33.0)	15 (43)

Taking tumor dormacy into account [43] looking for recurrances during a high risk time period and earlier detection might enable earlier targeted therapies [44]⁠ with a possible beneficial effect on the OAS. But clinically the question is when to look where and how. With new methods like liquid biopsy published [25, 45] the perspective for patients might become better. Yet those methods are neither widely available nor well evaluated. And despite the unavailability for most caretakers the cost and benefit is still unknown. Generally earlier detection of a recurrance has not been published to be associated with better survival rates [19]. In order to do so retrospectively more details regarding the recurrance need to be gathered in tumor databases (i.e. location, diameter, genetic profile, …). Here the combination of the BRENDA-Score with the intrinsic subtypes helps to identify the subgroup of patients worth monitoring closely.

## Conclusion

With a risk distribution per primary tumor data the clinician can individualise the follow up according to the BRENDA-Score. Available imaging technology or laboratory parameters can focus on the organ at risk. Liquid biopsy markers may be used at the optimal time for earlier detection of the recurrance. And of course tumor documentation needs to include more details regarding size, location and number of recurrances in order to proof a benefit of earlier detection.

The BRENDA-Score is only a starting point and the authors encourage everyone to take advantage of it.

## Data Availability

Upon written request to the BRENDA study group.

## Code availability

N/A.
